# Decoding the Architecture of Molecular Diodes: Rational Design for Ideal Rectification

**DOI:** 10.3390/molecules30142998

**Published:** 2025-07-17

**Authors:** Sara Gil-Guerrero, Nicolás Ramos-Berdullas, Marcos Mandado

**Affiliations:** Department of Physical Chemistry, University of Vigo, Lagoas-Marcosende s/n, 36310 Vigo, Spain; sgg@uvigo.es (S.G.-G.); nicolas.ramos@uvigo.es (N.R.-B.)

**Keywords:** molecular electronics, molecular rectifier, computational chemistry, electron deformation orbitals

## Abstract

The design of nanoscale electronic components remains a major challenge because we have limited control over the chemical and physical properties of their molecular constituents. Even subtle structural or compositional modifications can significantly alter their electronic behavior. Consequently, updating a molecular component often necessitates developing a new model from scratch. In this study, we present a comprehensive analysis of the rectification properties of a promising molecular diode initially proposed by Aviram and Van Dyck. The model has been systematically decomposed into fundamental building blocks, enabling the electron transport process to be examined both as an integrated event and as a sum of cooperative interactions. Our findings reveal that certain motifs—such as the D-σ-A architecture—play a significant role in rectification. However, achieving high-performance molecular rectifiers also requires cooperative interplay with other structural elements that contribute to rectification, such as asymmetric molecule–metal contacts. In this study, we conduct a detailed investigation of the roles these elements play in shaping the rectifying characteristics, and we further interpret their effects by analyzing the dominant transport channels under forward and backward bias conditions. This deeper understanding of the transport mechanism offers greater control over the system and opens the door for rational design strategies for improving rectification efficiency in future molecular devices.

## 1. Introduction

The miniaturization of electronic devices is one of the major goals of this century, with current advancements reaching the nano and molecular scales [1,2,3,4,5]. Achieving this objective, however, brings with it the challenge of exercising precise control over the properties of nanoscale and molecular systems to ensure they can perform their intended functions. Consequently, one of the primary aims in the field of molecular electronics is to understand the transport behavior of molecular junctions in relation to both the chemical characteristics of their constituent molecules and their physical response to electron flow. In this context, theoretical and computational modeling techniques have proven to be powerful tools for gaining insight into the processes that occur within molecular electronic components [6,7,8,9].

Among these components, molecular rectifiers pose a particular challenge for miniaturization [10,11,12,13,14,15,16,17,18,19,20,21,22,23,24,25,26,27,28,29,30,31,32,33,34,35,36]. Rectifiers act as one-way switches for the electron current, allowing flow in only a single direction. Therefore, enhancing the electron transport capacity of a rectifier must not compromise the inherent asymmetry that enables this unidirectional behavior. Pioneers in this area, Aviram and Ratner, introduced the concept of the “True Unimolecular Rectifier” (TUR) [10]. A TUR is a single-molecule rectifier whose asymmetric electron transport stems from an uneven distribution of frontier orbitals, resulting from a specific molecular architecture. According to their proposal, a rectifier’s structure must include an electron-donating fragment (D) and an electron-withdrawing fragment (A), connected via a bridge—either saturated (D-σ-A) or conjugated (D-π-A). The effectiveness of this design was attributed to the asymmetric alignment of the molecule’s frontier orbitals relative to the Fermi level of the metal electrodes.

However, in some systems, this static view of orbital distribution fails to account for the effects of an applied external electric field [11,16,26]. Such perturbations can alter not only the orbitals of the isolated molecule but also their alignment relative to the energy levels of the metal electrode. Thus, Metzger and co-workers highlighted the rarity of true TURs in practical systems [16]. Building upon this foundation, Van Dyck and Ratner proposed a more comprehensive rectifier model roughly a decade ago [27]. Their approach retained the TUR scheme, known as “U mechanism”, while incorporating an additional asymmetry-based anchoring strategy, termed the “S mechanism”. In this design, the D-σ-A architecture includes asymmetric anchoring groups: the donor fragment is a conjugated structure with a thiol anchor, and the acceptor is another conjugated structure, anchored via a cyanide group. The S mechanism introduces asymmetry in the electron transport through Schottky barriers [37], which are potential energy barriers formed at the molecule–metal interface. The presence of asymmetric linkers leads to an uneven spatial charge density at the two ends of the junction, a phenomenon known as the polarization effect. As a result, this dual approach enables control over frontier orbital alignment (via the U mechanism) as well as asymmetry in the interface barriers (via the S mechanism).

Other important external factors that can significantly affect the direction and magnitude of rectification—such as electrolyte concentration, intermolecular van der Waals forces, solid back/edge-gating, and liquid ion gating—have been previously investigated at the experimental level [38,39], but were not considered in this study due to the complexity involved in incorporating them into the electron transport computational simulations and our focus on factors intrinsic to the structure of the single-molecule junction. Therefore, in this work, we analyze the characteristics of the Van Dyck and Ratner rectifier using a conceptual framework analogous to the “Design for Disassembly” principle in architecture—a design approach that anticipates future modifications or dismantling. Here, the rectifier is evaluated based on its constituent building blocks: the single molecule, the electrode, and the molecule–metal contact. This approach allows for potential improvements in rectification efficiency through subtle structural modifications.

However, it is essential to ensure that such modifications do not significantly alter the alignment between the molecular electronic levels and the electrode Fermi level, as this could compromise rectification. Ideally, the Schottky barriers should remain stable and insensitive to changes in either the molecule or the metal—an effect known as Fermi-level pinning [40]. Strong coupling between the molecule and metal can override this stability, as the electronic levels of the junction become dominated by the molecule–metal interaction. Therefore, our analysis considers both the rectifier’s sensitivity to structural modifications and the strength of the molecule–metal coupling.

## 2. Results and Discussion

As mentioned in the introduction, the individual components of the molecular rectifier model proposed by Van Dyck and Ratner—including the electrode—have been evaluated separately to reveal the specific role each plays in the device’s overall performance.

### 2.1. The Molecular Moiety

**Figure 1:**
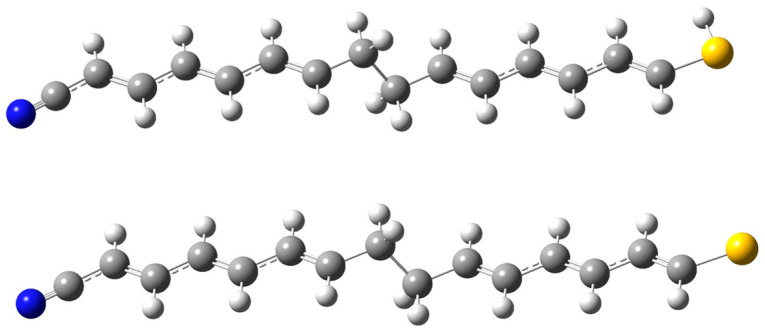
Structures of the protonated and deprotonated/radical forms of the isolated molecular rectifier with an ethyl σ-bridge. The blue, gray, white and yellow balls represent nitrogen, carbon, hydrogen and sulfur atoms, respectively.

The existence of a pure TUR has been questioned by some authors in the literature [11,16], prompting us to first investigate the rectification ability of the isolated molecular moiety. In this study, different structural forms of the molecule have been analyzed: the neutral molecule, in which the thiol group remains protonated; the anionic form, where the thiol group is deprotonated; and the radical form, resulting from the homolytic cleavage of the thiol hydrogen. The structures of the protonated and deprotonated/radical forms of the molecule with an ethyl σ-bridge are shown in Figure 1. Experimental studies on thiol–gold interactions have shown that adsorption onto gold surfaces leads to S–H bond dissociation [41]. Consequently, once integrated into the molecular junction, the molecule is likely to exist either in a negatively charged or radical state.

**Figure 2:**
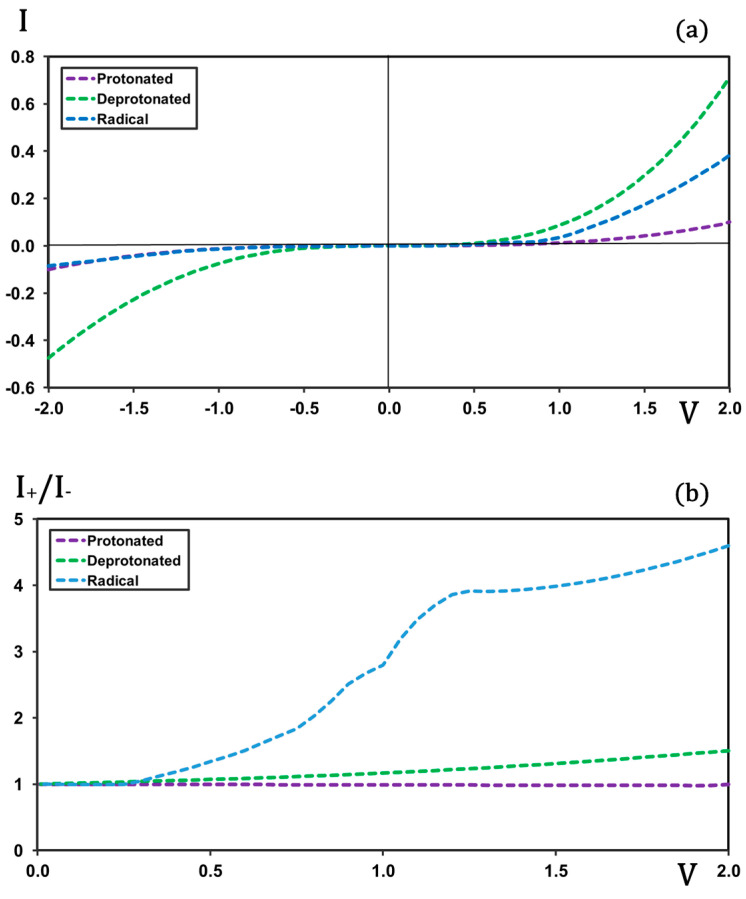
I-V (plot (**a**)) and rectification (plot (**b**)) profiles for the protonated, deprotonated and radical forms of the isolated molecular rectifier (without electrodes). Current in μA and voltage in V.

In the Figure 2a, the current–voltage (I-V) profiles obtained for the protonated, deprotonated, and radical forms of the molecule are compared. As observed, the protonated form exhibits the lowest current intensity and displays a largely symmetric profile with respect to forward and backward biases. This symmetry leads to a non-rectifying behavior, as reflected in the Figure 2b. In contrast, the radical form shows a significantly higher current at forward bias while maintaining an almost identical profile at backward bias. This asymmetry in the I-V characteristics results in a certain degree of rectification, although the rectification ratios are much lower than those predicted in previous calculations for the full molecular junction [36]. The deprotonated form also exhibits some asymmetry in its I-V profile—albeit to a lesser extent than the radical—showing slightly higher current intensities at forward bias. Notably, the overall current intensities for the deprotonated form are considerably higher than those for the radical form; the values are almost doubled. This makes the deprotonated species, a priori, the most suitable candidate for further analysis involving metal electrodes. Moreover, the radical form is chemically unstable and therefore difficult to handle under experimental conditions. In addition, the calculations for the isolated radical under applied bias showed significant convergence problems, which are evident in the irregular shape of the rectification-versus-voltage profile. For all these reasons, the study of the complete molecular junction has been carried out using the deprotonated form of the molecule.

### 2.2. The Central Sigma Unit

**Figure 3:**
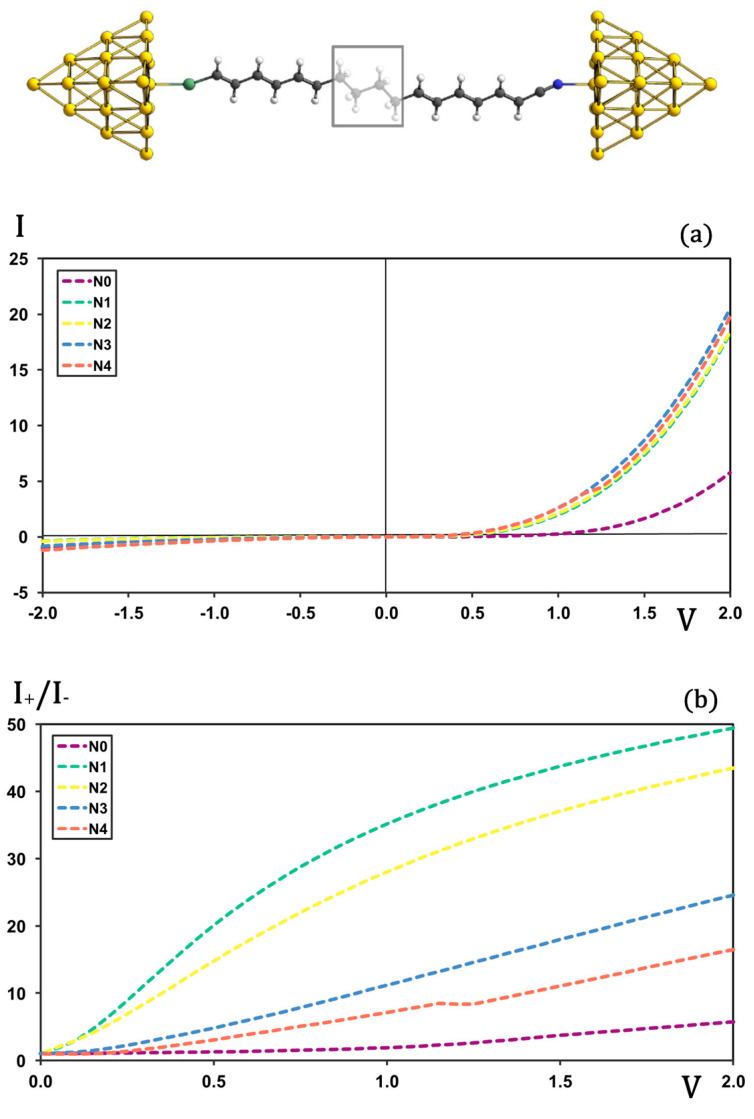
I-V (plot (**a**)) and rectification (plot (**b**)) profiles for molecular junctions with N ethyl units as a sigma bridge. Current in μA and voltage in V.

The next step in our analysis was to determine how the length of the σ-bridge affects rectification. The original molecular rectifier proposed by Van Dyck and Ratner employs a butyl linker between the electron–donor and electron–acceptor π-conjugated fragments [27]. In the Figure 3a, we compare the I-V characteristics of this prototype junction with those of analogues containing ethyl, hexyl, and octyl σ-bridges, as well as a derivative without any bridge. When a σ-bridge is present, the I-V profiles are nearly superimposable and, rather surprisingly, the current rises slightly with increasing bridge length under both forward and backward biases.

Although this rise is small, it occurs along the nominally non-conducting bias polarity and therefore has a pronounced impact on the rectification ratio (see Figure 3b). The rectification ability decreases with the length of the σ-bridge, following the order N1 > N2 > N3 > N4, with N1 outperforming N4 by more than a factor of three. A distinct loss of rectification is also observed upon lengthening the bridge from ethyl/butyl to hexyl/octyl.

Removing the σ-bridge altogether produces a marked drop in current along the conducting direction, leading to rectification ratios roughly an order of magnitude lower than those of the best-performing system (N1). In this bridge-free case, rectification originates solely from the Schottky barriers at the molecule–metal contacts—the so-called S mechanism—which yields rectification factors of about five at high bias. Therefore, the TUR scheme is essential, as the σ-bridge is responsible for inducing the asymmetric charge density distribution that enables directional charge transport.

We have further investigated the effect of enlarging the σ-bridge on the alignment of the frontier MOs with respect to the metal Fermi level. According to the Aviram–Ratner model [10], one might expect that this modification would alter the electronic levels of the molecule in such a way that the positions of the frontier orbitals no longer allow alignment with the Fermi level of the metal cluster. In this context, the relative positions of these orbitals are presented in Table 1. The orbital energies of both the gold cluster and the molecules indicate that the modification of the σ-unit does not lead to a drastic change in the relative positions of the molecule’s frontier orbitals with respect to the Fermi level. While these systems would be expected to exhibit similar rectification behavior, the results show, as mentioned before, a significant decrease in rectification ratios as the size of the σ-unit increases.

When analyzing the HOMO-LUMO gaps of the molecular junctions (Table 1), a clear relationship emerges—starting from the molecule containing the shortest σ-unit—between the decreasing rectification ratios and the narrowing of the energy gap. The gaps also explain the drop in the rectification ratios when lengthening the bridge from ethyl/butyl to hexyl/octyl. This further demonstrates that, in covalently coupled systems, the behavior cannot be fully understood by examining the isolated molecular electronic structure alone. Instead, the features responsible for variations in transport properties arise from the full molecular junction as an integrated system.

### 2.3. The Molecule–Metal Contact

**Figure 4:**
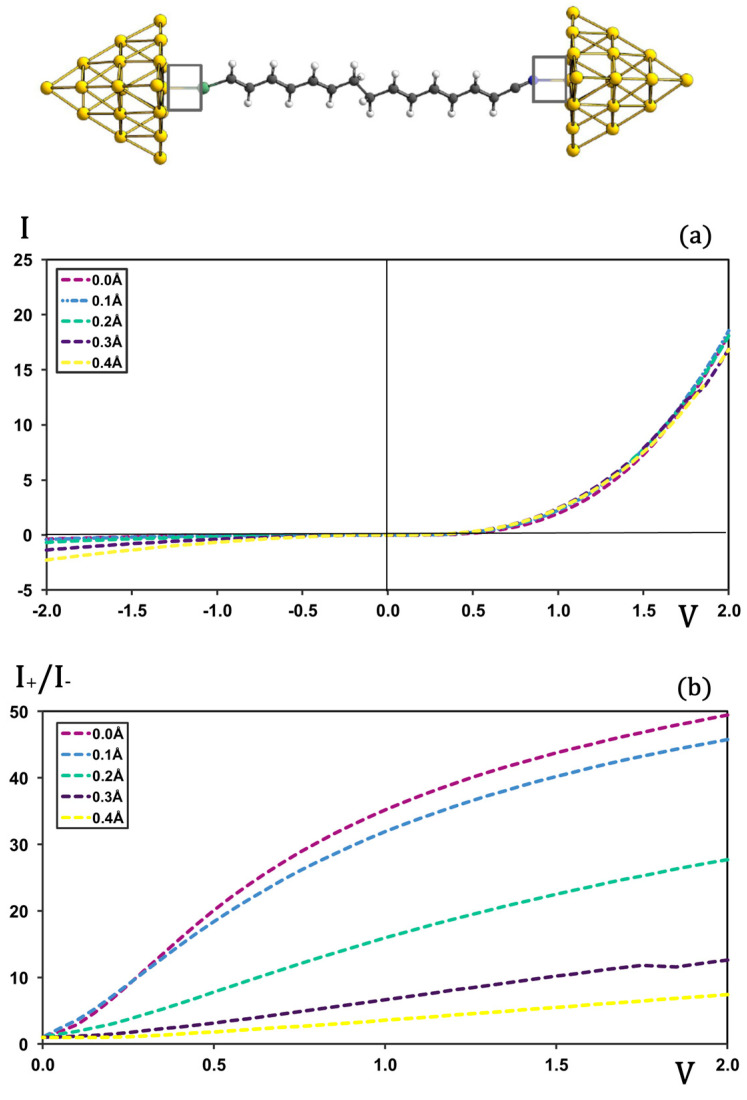
I-V (plot (**a**)) and rectification (plot (**b**)) profiles for molecular junctions with S-Au and N-Au contacts elongated from 0.0 to 4.0 Å. Current in μA and voltage in V.

To assess how the molecule–metal contact influences rectification, we examined the I-V characteristics and rectification ratios of junctions in which the S–Au and N–Au bond lengths were either elongated (Figure 4) or shortened (Figure S1) with respect to their equilibrium values in the best-performing rectifier (N1).

When the contacts are stretched, the forward-bias current remains essentially unchanged over elongations of 0.0–0.4 Å (see Figure 4a). By contrast, the backward-bias current grows markedly, in relative terms, with increasing contact length, causing rectification ratios to drop steeply for elongations ≥ 0.2 Å (see Figure 4b).

Shortening the S-Au and N-Au distances yields a different picture: both forward- and backward-bias I-V profiles remain nearly identical to those at equilibrium (Figure S1a), so the rectification ratios decrease only slightly (Figure S1b).

Overall, these observations underscore the pivotal role of the covalent S-Au bond in dictating rectification. Partially weakening this covalent interaction by stretching the contact—and thereby enhancing its electrostatic character—significantly degrades rectification. Conversely, compressing the contact leaves the covalent nature largely intact, and rectification performance is maintained to a great extent. The same can be inferred for the N-Au contact on the opposite side of the junction; however, in this case, the covalent character of the bond is expected to be weaker than that of the sulfur link.

### 2.4. The Metal Electrode

As a final element in the analysis of the Van Dyck–Ratner rectifier, we evaluated the effect of substituting gold electrodes with silver, a metal with higher bulk conductivity and a lower work function. The geometry optimization of the Ag-based junction followed the same protocol used for the Au system (see the Section 3 for details). The typical S-Ag and N-Ag bond lengths are slightly longer than their S-Au and N-Au counterparts—by approximately 0.1–0.2 Å in both cases. Moreover, the bonds formed with gold are not only stronger, exhibiting higher bond dissociation energies, but also more covalent compared to those formed with silver, which tend to be more ionic or electrostatic.

**Figure 5:**
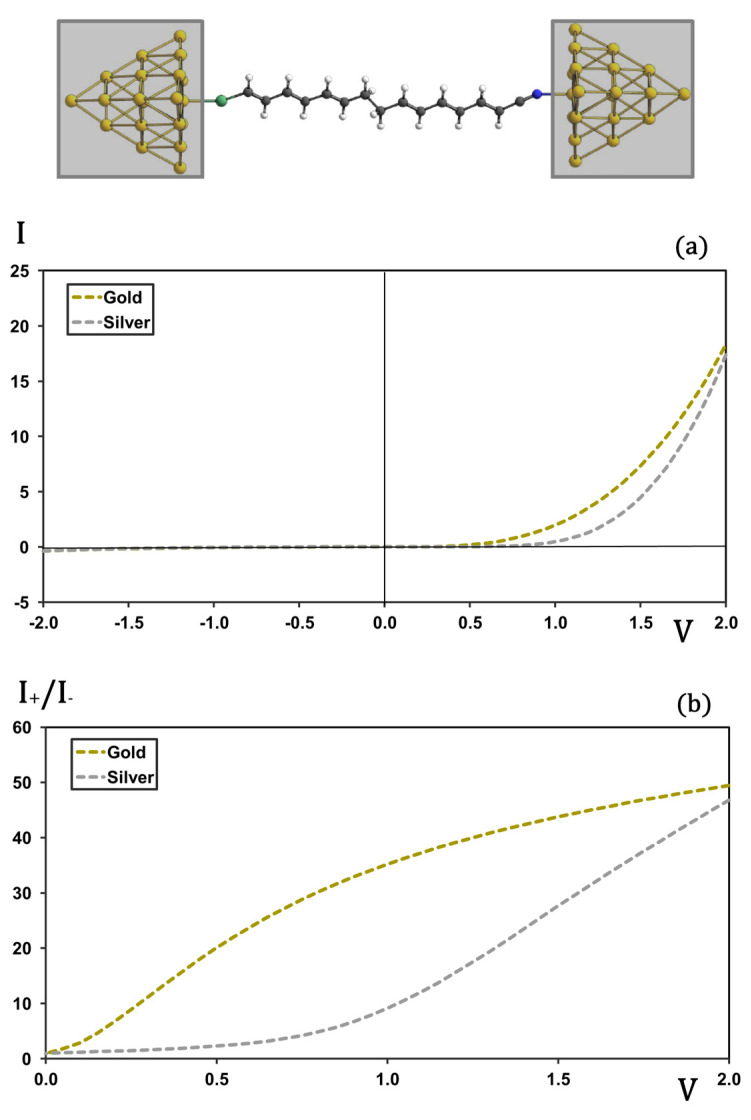
I-V (plot (**a**)) and rectification (plot (**b**)) profiles for molecular junctions formed with gold and silver electrodes. Current in μA and voltage in V.

Figure 5 compares the I-V characteristics and rectification ratios for the N1 molecule bound to Au and Ag cluster electrodes. In the reverse-bias regime (plot (a)), both Au and Ag junctions exhibit negligible current. Pronounced differences emerge under forward bias between 0.5 V and 1.5 V: for Au electrodes, the current begins its monotonic rise at ~0.4 V, whereas for Ag electrodes the onset of appreciable current is delayed until ~0.8 V. In the plot (b), rectification ratios are compared for the two metals. While gold exhibits significant rectification across the entire voltage range, silver shows negligible rectification until the applied bias exceeds 0.8 V. Beyond this point, rectification in the silver-based junction increases sharply, eventually reaching values comparable to those observed with gold electrodes in the high-voltage regime.

This shift in the turn-on voltage for silver can be attributed to differences in the nature of the molecule–metal contact. As mentioned previously, the S-Au and N-Au bonds are stronger and exhibit a more covalent character. This covalency facilitates stronger electronic coupling between the molecule and the gold electrode, promoting more efficient charge injection at lower bias voltages. In contrast, the S-Ag and N-Ag bonds are expected to be more ionic or electrostatic, resulting in weaker effective coupling and reduced mixing between molecular and electrode electronic levels. These factors contribute to the observed delayed current onset and suppressed rectification in the silver junction at low bias. At higher voltages, where the potential barriers at the electrode–molecule interface are more easily overcome, electron transport in silver becomes more efficient, consistent with its higher bulk conductivity and lower work function.

### 2.5. Transport Channels

As described in detail in the theoretical section of the Supplementary Materials, the total conductance of the junctions is computed as the sum of contributions from individual transport channels, each formed by a combination of a hole and an electron function—referred to as EDOs. These EDOs emerge from the bias-induced mixing of occupied and virtual molecular orbitals (MOs). Analyzing the dominant conducting channels can yield valuable insights into the origin of the differing rectification behavior observed [36].

**Figure 6:**
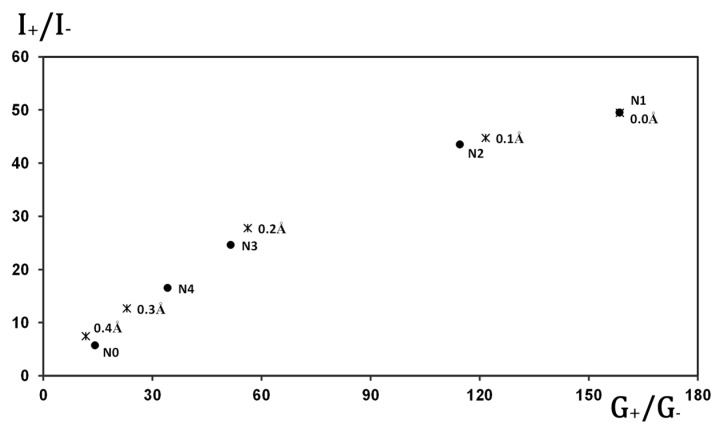
The ratio between the forward and backward conductance (G_+_/G_−_) for the dominating channel versus the rectification ratio obtained at ±2 V for the different molecular junctions studied.

Figure 6 provides evidence that the rectification behavior of the molecular junctions can be qualitatively inferred by analyzing only the dominant transport channel. In this plot, the rectification ratio at 2 V is shown for the various gold-based junctions studied, plotted against the ratio of forward to reverse conductance for their respective main transport channels. A clear one-to-one correlation is observed, indicating that the rectifying character can be effectively captured by examining the asymmetry in conductance of the primary channel alone.

**Figure 7:**
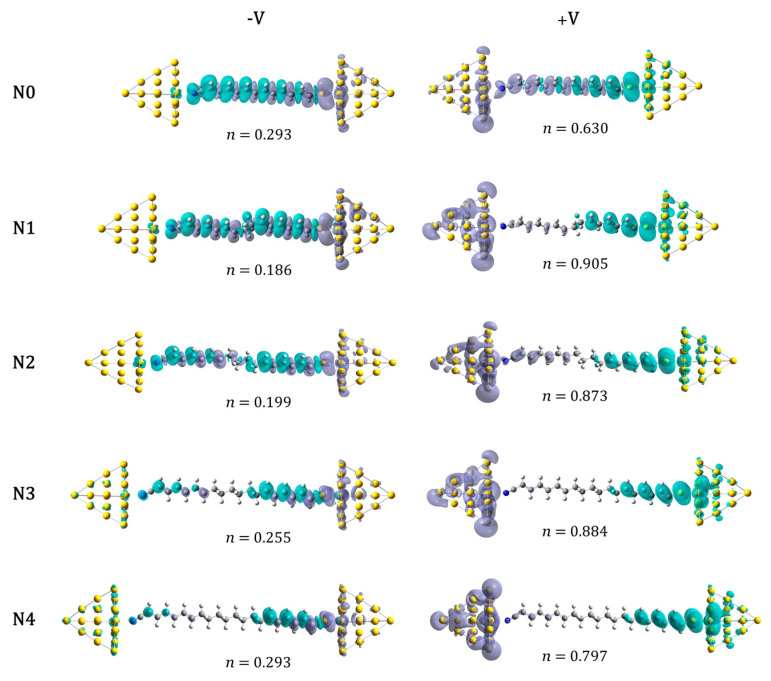
Forward and backward main conducting channels obtained for gold-based junctions with σ-bridge units of different lengths under a bias of ±2 V. N0 (no σ-bridge), N1 (ethyl), N2 (butyl), N3 (hexyl) and N4 (octyl). Isosurface value of 2 × 10^−4^.

Figure 7 displays the dominant transport channels obtained at ±2 V for junctions containing σ-bridging units of different lengths; the weight of each channel, given by the eigenvalue of the associated EDOs, is indicated alongside each plot. Under the forward bias, the contrast between systems N0 and N1 is pronounced. In N1, the leading channel supports a clear electron flux from the donor toward the acceptor side, whereas in N0 it is dominated by internal charge polarization confined to the ethylene moieties, lending a more dielectric character to the junction. In the backward bias direction, the primary channels in N0 and N1 are qualitatively similar: both are governed by strong internal polarization, which accounts for the very low conductance observed under reverse bias.

For longer σ-bridging units (N2, N3, N4) the transport channels remain qualitatively similar to those of N1. Because the same isosurface value is applied in every representation, the visible surface area decreases as the molecular length grows. Here, the key change lies in the channel weights: the weight of the forward-bias channel diminishes with increasing σ-bridge length, whereas the weight of the backward-bias channel rises. A comparable trend appears in the dominant channels of junctions whose molecule–metal distances are elongated relative to equilibrium (Figure S2), although in that case only the backward-bias channel shows a pronounced weight increase. By contrast, shortening the contacts leaves both channel weights virtually unchanged, which accounts for the modest variations in rectification when the molecule–metal contacts are shortened.

Figure S3 compares the channels calculated at ±2 V and ±0.7 V for gold- and silver-based junctions. The most significant differences arise in the forward-bias direction, particularly at 0.7 V. In the Au junction, the hole function is localized on the metal cluster, whereas in the Ag junction it is concentrated in the molecule’s donor region. The forward-bias channel weight is likewise substantially lower for silver than for gold. In line with the discussion in Section 2.4, these observations indicate a reduced electron transfer ability from the electrode to the molecule in the Ag junction at low voltages. This is attributed to weaker electronic coupling between the molecule and electrode compared to the Au junction, leading to a shift in rectification onset toward higher voltages.

However, a thorough explanation of the differences observed between metal electrodes would require a more extensive study involving a broader set of molecular rectifiers and a wider range of metal electrodes and their combinations, which lies beyond the scope of the present work.

## 3. Computational Details

The original rectifier studied by Van Dyck and Ratner consisted of a linear molecular chain composed of two hexatriene units connected by a butyl bridge, terminated with a thiol group at one end and a cyano group at the other. The rectification behavior of this molecule was theoretically evaluated in a molecular junction with gold electrodes [27,36]. To investigate the influence of the various molecular components on rectification performance, we initially studied the protonated, deprotonated, and radical forms of the molecule. The second and third forms correspond, respectively, to the thiolate anion and the radical formed upon the homolytic rupture of the S-H bond. In addition, we explored variations of the molecular chain in which the butyl bridge (N2) was replaced by alkyl chains of different length—ethyl (N1), hexyl (N3), and octyl (N4)—and one structure in which the bridge was entirely removed (N0). We further examined molecular junctions formed with silver electrodes to assess the effects of the electrodes. To further investigate the influence of the molecule–electrode contacts on the rectification ratio, the molecule–metal distances were systematically varied by ±0.4 Å from their equilibrium values, in increments of ±0.1 Å.

Geometry optimizations and wavefunction calculations were performed using density functional theory (DFT) as implemented in the Gaussian 16 program [42]. The energy minima of the molecules were obtained using the B3PW91 functional and the 6-311G(d, p) basis set. The metal electrodes were modeled as pyramidal gold or silver clusters composed of twenty atoms. Their geometries were optimized using the same functional, in conjunction with a double-zeta basis set and the LANL2DZ pseudopotential to describe valence and core electrons, respectively.

Subsequently, electrode–molecule–electrode (sandwich-like) complexes were assembled, as illustrated in Figure 1, and the molecule–metal distances were optimized. Wavefunctions for these complexes—both with and without applied external bias—were calculated at the same theoretical level as used for the isolated fragments. The methodology used to calculate electric current and conductance is based on the time–energy uncertainty principle combined with the definition of transport channels constructed from electron deformation orbitals (EDOs) [43,44,45,46,47]. A detailed theoretical section is provided in the Supplementary Materials. Conductance and transport channel calculations were performed using an in-house implementation of the FIOs-EDOs v2022.1 software [48], with the transport channels subsequently visualized via the GaussView 5.0.8 graphical interface [49].

Given the near-linear configuration of the metal–molecule–metal junctions, an external electric field of appropriate intensity was applied along the axis defined by the central atoms of the opposing metal clusters to simulate the bias voltage. Thus, voltages ranging from −2 V to +2 V were applied to the junctions in ±0.05 V increments to obtain the conductance (G) and the current/voltage (I-V) profiles, as well as the corresponding rectification ratios (I_+_/I_−_).

## 4. Conclusions

This study presents a comprehensive analysis of the individual components governing the rectification behavior in a molecular junction based on the Van Dyck–Ratner rectifier model. By systematically dissecting the roles of the molecular moiety, σ-bridge, molecule–metal contacts and metal electrodes, we have identified the fundamental mechanisms responsible for directional electron transport.

Our findings confirm that rectification cannot be attributed solely to the intrinsic electronic asymmetry of the isolated molecule. Instead, effective rectification arises from the interplay between molecular structure and junction properties. Among the molecular forms analyzed, the deprotonated species emerged as the most suitable candidate due to its chemical stability and higher current output. The σ-bridge, while it does not significantly alter the frontier orbital alignment, plays a crucial role in sustaining asymmetric charge distribution; longer bridges compromise this asymmetry, leading to diminished rectification performance.

We have also shown that molecule–metal contact distances have a substantial impact: stretching these contacts weakens covalent interactions and undermines rectification, while shortening them has minimal effect. Substituting gold with silver electrodes results in a higher rectification onset voltage, reflecting a weaker molecule–electrode coupling despite similar bonding motifs. Transport channel analysis further revealed that rectification behavior can be qualitatively predicted from the asymmetry in the conductance of the dominant channel, validating its utility as a diagnostic tool for molecular electronic devices.

Altogether, these insights emphasize that the rectification properties of molecular junctions derive from a concerted contribution of molecular, interfacial, and electrode factors. This underscores the importance of evaluating the full junction architecture, rather than isolated components, when designing functional molecular electronic devices.

## Figures and Tables

**Table 1:** Energy values of the frontier orbitals of both the pyramidal gold cluster and the isolated molecules with σ-bridges of different length, and HOMO-LUMO energy gaps for the corresponding molecular junctions (MJs). N0 (no σ-bridge), N1 (ethyl), N2 (butyl), N3 (hexyl) and N4 (octyl). Energies in Hartrees and gaps in eV.

	Au-Cluster	N0	N1	N2	N3	N4
HOMO	−0.1266	−0.2028	−0.2489	−0.2462	−0.2447	−0.2438
LUMO	−0.2331	−0.1067	−0.0564	−0.0554	−0.0548	−0.0546
Gap (MJ)		1.04	0.29	0.24	0.13	0.11

## Data Availability

The original contributions presented in this study are included in the article/Supplementary Materials. Further inquiries can be directed to the corresponding author.

## Supplementary Materials

The following supporting information can be downloaded at https://www.mdpi.com/article/10.3390/molecules30142998/s1, Theoretical Background, Figure S1: I-V and rectification profiles for molecular junctions with S-Au and N-Au contacts shortened from 0.0 to 4.0 Å; Figure S2: Forward and backward main conducting channels obtained for gold-based junctions at equilibrium S-Au and N-Au distances and elongated and shortened 4.0 Å under a bias of ±2 V; Figure S3: Forward and backward main conducting channels obtained for gold-based and silver-based junctions under biases of ±0.7 V and ±2 V.
