# Supplementary material for: Decoding the Architecture of Molecular Diodes: Rational Design for Ideal Rectification
Source: Molecules. 2025 Jul 17;30(14):2998. doi: 10.3390/molecules30142998 (PMC12300316; doi:10.3390/molecules30142998)
Supplement: Supplementary file 1 [file molecules-30-02998-s001.zip › molecules-3741384-supplementary.pdf]

Supplementary Materials

Decoding the Architecture of Molecular Diodes: Rational Design for Ideal Rectification

Sara Gil-Guerrero, Nicolás Ramos-Berdullas, Marcos Mandado

Department of Physical Chemistry, University of Vigo, Lagoas-Marcosende s/n, 36310 Vigo, Spain

1. Theoretical background

The methodology employed in this study to calculate electrical conductance is based on the concept that, analogous to the quantum conductance formulation derived from the time–energy uncertainty principle [1,2], Heisenberg's uncertainty principle can also be leveraged to obtain a straightforward and computationally efficient expression for electric current. This approach was initially proposed by Ortiz and Seminario [3] and later refined by Ramos and Mandado [4]. Accordingly, using the time–energy uncertainty relation, the electric current can be expressed as,

$$I = \frac{\Delta E_{def} \Delta q_{E-E}}{h} \quad \text{(S1)}$$

Here, Δq_{E-E} denotes the electronic charge transferred between the electrodes under the influence of an applied bias voltage, while ΔE_{def} represents the deformation energy, i.e., the energetic cost of transferring this charge against the internal molecular potential. The simplicity of Equation (S1) lies in its reliance solely on the calculation of energies and wavefunctions for the unperturbed and electrically perturbed states of the electrode–molecule–electrode system. However, its main drawback is the need to model the electrodes with a sufficiently large number of atoms to achieve quantitatively meaningful results, as the molecular junction is treated as a single block. Furthermore, this initial approach does not offer detailed insights into the underlying electron transport

mechanisms, but merely yields current values. For these reasons, the practical utility of Equation (S1) may appear limited at first glance.

Nonetheless, a recent study has demonstrated that the electrical conductance, obtained as the derivative of Equation (S1) with respect to the applied bias voltage, can be decomposed into contributions from distinct electron transport pathways, referred to as transport channels. Notably, the resulting expression presented in reference [5] aligns with the well-established Landauer–Büttiker formalism (see Equation (S1)), widely used in the literature, in which the conductance G is expressed as the product of the quantum of conductance and a transmission function.

$$G = \frac{2e^2}{h} \mathrm{Tr}(\mathbf{t}^\dagger \mathbf{t}) \tag{S2}$$

As shown in the equation above, the transmission function is calculated as the trace of the product of a matrix $\mathbf{t}$ and its transpose. The elements of this matrix are given by,

$$t_{ij} = \left(\int \mathrm{Tr}\left(\mathbf{\Theta}_i^\dagger \hat{r} \mathbf{\Theta}_i\right) d\tau \int_{\Omega_E} \mathrm{Tr}\left(\mathbf{\Theta}_j^\dagger \mathbf{\Theta}_j\right) d\tau \right) \tag{S3}$$

Here, $\mathbf{\Theta}$ represents the conducting channels responsible for electron transport. The second integral in Equation (S2) accounts for the charge transferred between the electrodes, corresponding to the minimum amount of charge either leaving or reaching an electrode at a given bias voltage. Each channel is mathematically described by the following complex matrix,

$$\mathbf{\Theta} = n_{\boldsymbol{k}}^{1/2} \begin{pmatrix} \xi_k^+ & 0 \\ 0 & i\xi_k^- \end{pmatrix} \tag{S4}$$

where, ξ_k^+ and ξ_k^- and represent a pair of electron deformation orbitals (EDOs), and n_k is the absolute value of the occupation number associated with that pair. EDOs were introduced as a molecular orbital decomposition of the electron density response of molecular systems to a constant electric perturbation. Thus, when the polarization density induced by the electric perturbation is expressed in the basis of the unperturbed molecular

orbitals (MOs), it can be represented through a polarization (or deformation) density matrix. The EDOs and their corresponding occupation numbers are then obtained as the eigenfunctions and eigenvalues, respectively, resulting from the diagonalization of this matrix.

Because the unperturbed MOs form an orthonormal set and the trace of the polarization density matrix is zero, the EDOs naturally arise in pairs with equal magnitude but opposite sign eigenvalues. Each such pair consists of an electron-like orbital (ξ_k^+) and a hole-like orbital (ξ_k^-), both of which are constructed as linear combinations of the set of unperturbed occupied and virtual MOs (χ_j).

$$\xi_i = \sum_j u_{ij}\chi_j \qquad \text{(S5)}$$

Based on Equations (S3)-(S5), electron transport can be understood in terms of the mixing between the occupied and virtual MO subspaces induced by the external bias voltage. Moreover, the electrical conductance can be correlated with the extent of electron promotion from occupied to virtual molecular orbitals, and certain symmetry rules were previously established using simple model molecules. This connection between conductance and occupied-to-virtual electron promotion is particularly noteworthy, as it aligns closely with the solid-state interpretation of electron transport. Other electric response properties, such as (hyper)polarizabilities, are also closely linked to the ease with which electrons can be promoted from occupied to virtual states.

Applying Equation (S2) for a range of voltages, the G-V curves can be represented, and the corresponding electric currents (*I*) obtained from the well-known relation between *G*, *I* and *V*.

$$G = \frac{dI}{dV} \qquad \text{(S6)}$$

In this work, we have employed the finite difference approximation to obtain the current for a given range of voltages, and then the I-V curves.

References

1. Batra, I. P. Origin of Conductance Quantization. *Surf. Sci.* **1998**, *395*, 43-45.
2. Batra, I. P. From Uncertainty to Certainty in Quantum Conductance of Nanowires. *Solid State Comm.* **2002**, *124*, 463-467.
3. Ortiz, D. O.; Seminario, J. M. Direct Approach for the Electron Transport Through Molecules. *J. Chem. Phys.* **2007**, *127*, 111106.
4. Ramos-Berdullas, N.; Mandado, M. Revisiting the Calculation of I/V Profiles in Molecular Junctions Using the Uncertainty Principle. *J. Phys. Chem. A* **2014**, *118*, 3827−3834.
5. Ramos-Berdullas, N.; Gil-Guerrero, S.; Mandado, M. Transmission Channels in the Time-Energy Uncertainty Relation Approach to Molecular Conductance: Symmetry Rules for the Electron Transport in Molecules. *Int. J. Quantum Chem.* **2018**, *118*, e25651.

2. Additional figures for transport channels

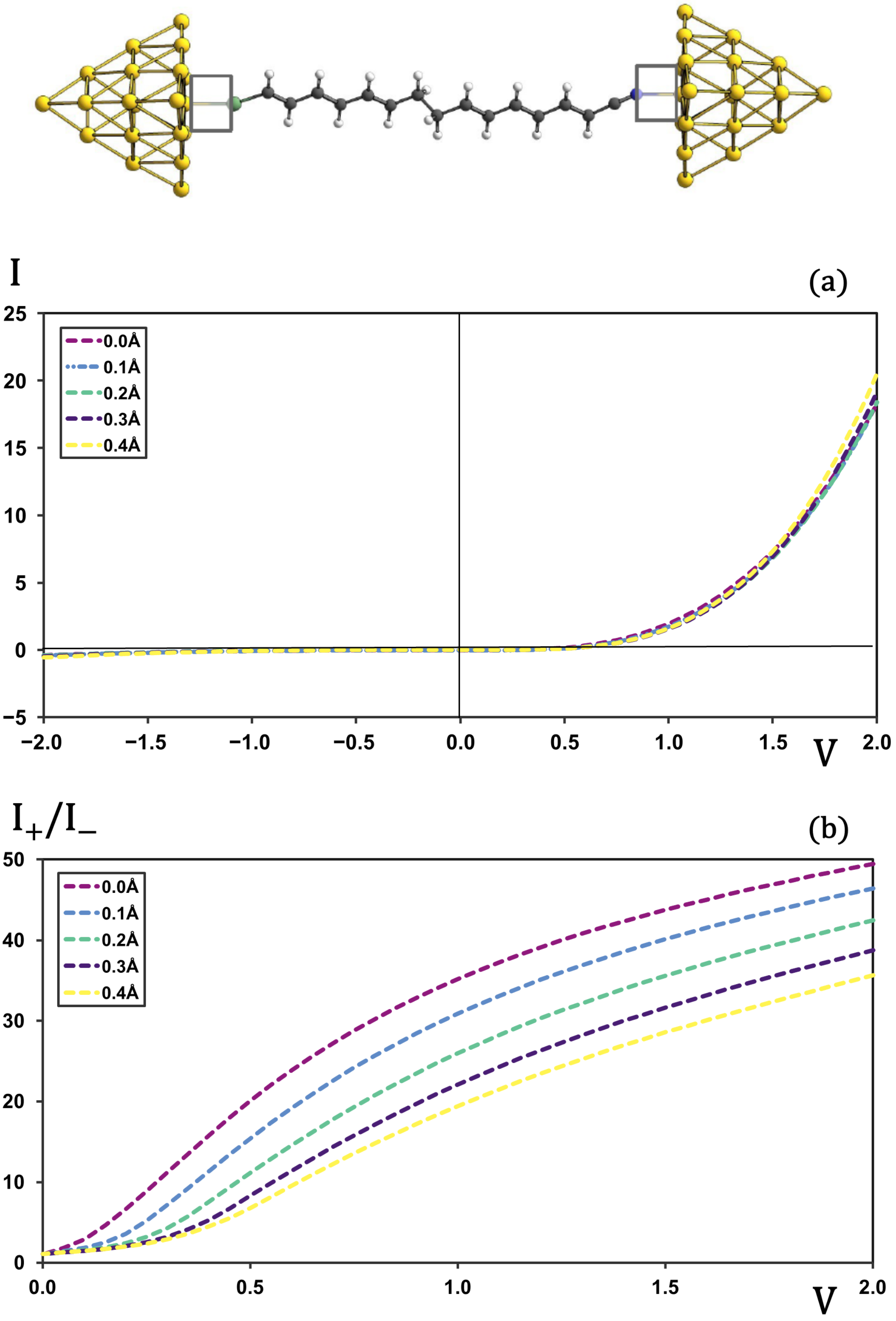

Figure S1: I-V (plot (a)) and rectification (plot (b)) profiles for molecular junctions with S-Au and N-Au contacts shortened from 0.0 to 4.0 Å. Current in μA and voltage in V.

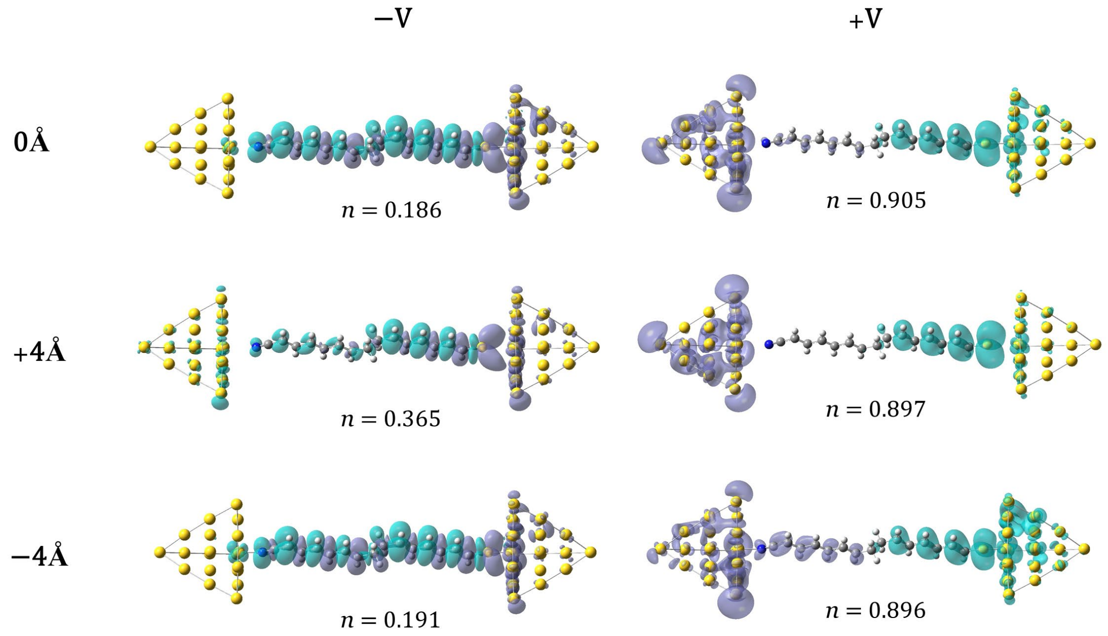

Figure S2: Forward and backward main conducting channels obtained for gold-based junctions at equilibrium S-Au and N-Au distances and elongated and shortened 4.0 Å under bias of ±2 V. Isosurface value of $2\cdot10^{-4}$.

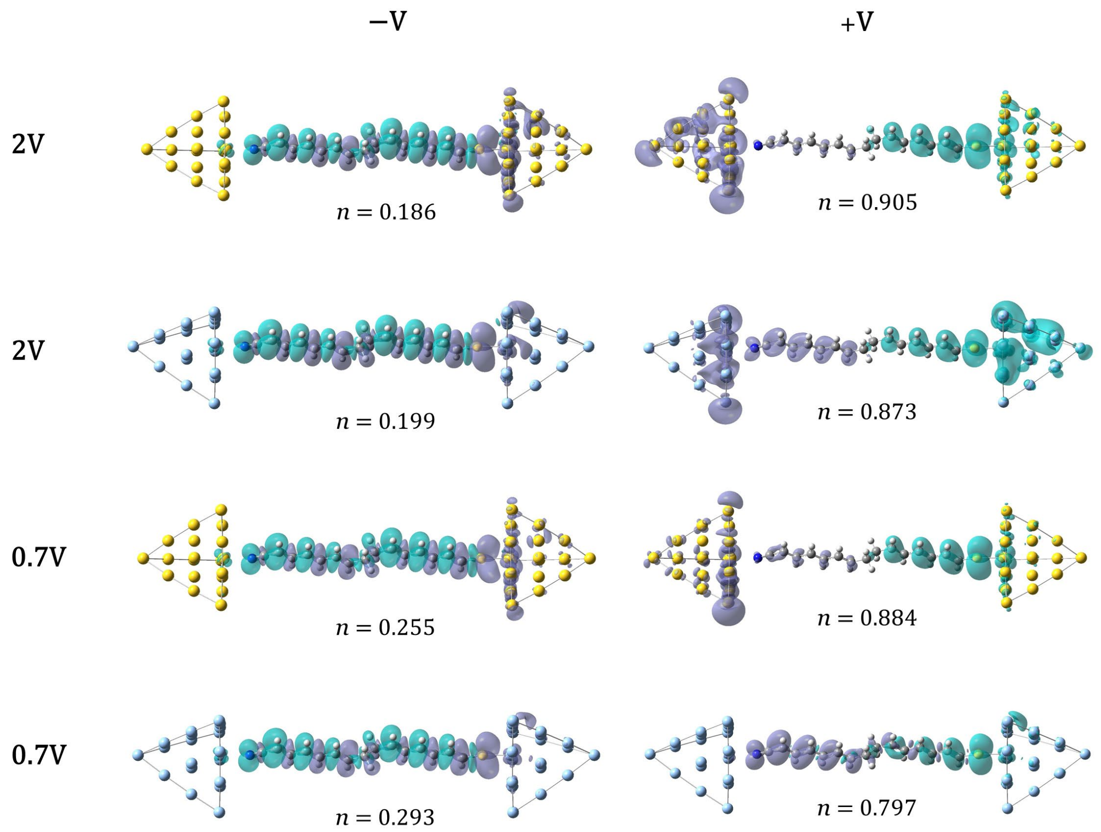

Figure S3: Forward and backward main conducting channels obtained for gold-based and silver-based junctions under bias of ±0.7 V and ±2 V. Isosurface value of $2 \cdot 10^{-4}$.
